# Mapping the Relationship Between Core Executive Functions and Mind Wandering in Children and Adolescents: A Systematic Review

**DOI:** 10.3390/jintelligence14020020

**Published:** 2026-02-01

**Authors:** Ioannis G. Katsantonis, Argyrios Katsantonis

**Affiliations:** 1 Department of Early Childhood Education and Education Sciences, School of Humanities and Social Sciences, University of Patras, 26504 Patras, Greece; 2 Department of Education Sciences and Social Work, School of Humanities and Social Sciences, University of Patras, 26504 Patras, Greece; up1083371@ac.upatras.gr

**Keywords:** mind wandering, off-task thoughts, executive functions, working memory, inhibitory control, cognitive flexibility, daydreaming, task-unrelated thinking, systematic review

## Abstract

Internationally, there are several studies that examined the relationship between core executive functions (working memory, inhibitory control, and cognitive flexibility) and mind wandering. These studies focused mostly on adult samples and there are fewer studies that examined this relationship with children and adolescent samples. Therefore, the current systematic review aims to identify and critically examine the existing peer-reviewed literature on the relationship between the core executive functions and mind wandering. Journal articles reporting quantitative results were identified through keyword searches in PsycINFO, Scopus, and PubMed. In total, 750 references were identified using the specified keywords. Among those, only ten studies were deemed to fit the inclusion criteria. The majority of the studies employed behavioural measures. The evidence on the relationship between the core executive functions and mind wandering was rather scarce and mixed. Most of the studies suggest that working memory capacity is critical for reduced mind wandering. The evidence regarding inhibitory control is rather mixed. Cognitive flexibility may underpin adaptive reallocation of attention between internal and external states, producing performance declines. The directional nature of the relationship between the three core executive functions and mind wandering is largely an unresolved matter, which requires further research.

## 1. Introduction

Mind wandering (MW) is a topic that has gained increased attention in cognitive science in recent decades (Callard et al., 2013). MW is an umbrella term that encompasses cognitive phenomena that describe self-generated mental activity (Smallwood, 2013). According to a recent study, the attention system has the tendency to focus inward on self-generated thoughts, diverting attention away from the requirements of the task one is preoccupied with (Zanesco et al., 2025). In the literature, MW has been examined under several terms, such as daydreaming, fantasising, task-unrelated or off-task thinking, and spontaneous thinking, among others (Callard et al., 2013). Although MW has huge potential in boosting creative thinking, there are several negative consequences of MW, such as the emergence of a worse mood and depression (Kawashima et al., 2023), increasing feelings of disengagement (e.g., boredom), and worse task performance (Zanesco et al., 2025). Worse task performance is a particularly noticeable product of MW, especially in cognitively demanding tasks (Keulers & Jonkman, 2019). MW is generally a difficult factor to examine because its unique nature poses methodological challenges in cognitive science (Gruberger et al., 2011). Therefore, the factors contributing to MW require further empirical research. Over recent years, several explanatory models and accounts of MW have been introduced in the literature, such as the sleep-drive view of MW in children (Spruyt et al., 2019), the “default-mode” network view (Gruberger et al., 2011), the executive-failure hypothesis (McVay & Kane, 2010a, 2010b), and the resource-control theory (Thomson et al., 2015). The current systematic review is focused on the latter two explanatory accounts.

Executive functions (EFs) is an umbrella term that refers to higher-order cognitive control processes, which permit individuals to maintain goal-oriented behaviour, to inhibit instinctual and/or irrelevant impulses, and flexibly shift between tasks and mental states (Miyake & Friedman, 2012). According to the seminal study by Miyake et al. (2000), the three core EFs are working memory (WM), inhibitory control, and cognitive flexibility. WM is essential for supporting the maintenance and processing of task-related goals (Baddeley, 2020). Inhibitory control regulates attention and suppresses prepotent and distracting stimuli during task performance (Kang et al., 2022) and cognitive flexibility allows individuals to switch between different and sometimes competing tasks and demands (Beckmann et al., 2024). In studies that employed adult samples, the evidence has pointed toward a robust negative association between MW and EFs (Biederman et al., 2019; Kane et al., 2016; Zavagnin et al., 2014). However, there are fewer studies that report on the relationship between MW and EFs with child and adolescent (up to 20 years old) samples. From our perspective, it is important to understand how MW is related to EFs in childhood and adolescence because these are two critical periods for cognitive development (Katsantonis, 2024; Kievit, 2020). In the next sections, we are going to present the theoretical framework for this review and outline our rationale for the importance of theoretically examining the relationship between EFs and MW in childhood and adolescence.

### 1.1. Mind Wandering: Definitions and Theoretical Explanations

MW is typically conceptualised as an attentional drift away from the ongoing processing of a task or environment toward internally generated mental content (Pelagatti et al., 2025). A study reported that MW instances occur during about 30% to 50% of humans’ waking hours (Zanesco et al., 2025). MW instances have been described in past research in terms of task-unrelated thinking (Gruberger et al., 2011; Keulers & Jonkman, 2019) or stimulus-independent thinking (Blondé et al., 2022; Gruberger et al., 2011). Contemporary works have decided that MW is best described as a continuum that can range from average task-unrelated thinking to maladaptive thinking that is associated with a mental disorder or executive dysfunction (Nazari et al., 2025). As a multidimensional construct, MW instances have been clustered as future- versus past-oriented (i.e., the mind drifts to past or future concerns), adaptive versus maladaptive outcomes (i.e., creative thinking versus feelings of boredom), and spontaneous versus deliberate MW (i.e., self-driven versus externally driven) (Nazari et al., 2025).

Gruberger et al. (2011) describe MW as an implicit cognitive phenomenon because it manifests without any external cues; it is sometimes unintentional and it presents methodological difficulties when trying to trace its origin and when replicating it. Evidence on the neural correlates of MW instances has been reported in recent decades. For instance, there is recent evidence showing that the “default mode network” (DMN) is associated with MW (Giacometti Giordani et al., 2023; Philippi et al., 2021). A higher activity within the DMN has been correlated with more instances of MW (Giacometti Giordani et al., 2023).

Several theoretical explanations of MW have been proposed in the literature (see (Smallwood, 2013) for review). One prominent hypothesis on the underlying mechanism of MW is called the ‘executive-failure hypothesis’ (McVay & Kane, 2010a, 2010b). The executive-failure hypothesis proposes that MW can be conceptualised as a lapse in cognitive control characterised by the inability to fixate attention on the task at hand (McVay & Kane, 2010b). The resource-control theory (Thomson et al., 2015), which subsumes key arguments of the executive-failure hypothesis, argues that cognitive control is a fundamental cognitive process that is a requirement for maintaining attention on the task and regulating the occurrences of task-unrelated thinking (Zanesco et al., 2025). Instead of solely attributing instances of MW to a failure of executive control, the resource-control theory maintains that MW is more likely to occur over time as cognitive resources are distributed to the task and task-unrelated thoughts (Zanesco et al., 2025).

Empirical research on the relationship between MW and EFs in adult samples has shown that older adults tend to report fewer instances of MW compared to younger adults, which is unexpected since cognitive function declines with age (Jackson & Balota, 2012; Robison et al., 2022). However, older adults might have a better capacity for cognitive control. Thus, the key question is what is the link between the development of EFs in children and adolescents and MW.

### 1.2. Development of Executive Functions in Childhood and Adolescence

The development of EFs in childhood and adolescence may play a moderating role in the relationship between the core EFs and MW. Thus, we need to consider the developmental trajectory of EFs from early childhood to adolescence. A recent empirical study that examined the developmental trajectory of EFs in a large sample spanning the ages of 8 to 35 years old reported rapid positive development from late childhood to middle adolescence (ages 10 to 15 years old) and stabilisation of the trajectory from middle to late adolescence (ages 18–20 years old) (Tervo-Clemmens et al., 2023). Another empirical study compared the three core EFs in adolescents (10–17 years old) to that of young adults (ages 18–29 years old) and adults (30–49 years old) (Ferguson et al., 2021). This study showed that inhibitory control and WM capacity were better in the young adult sample compared to the adolescent sample (Ferguson et al., 2021).

From a developmental perspective, the unity–diversity models of EFs could be useful for interpreting the association between EFs and MW. The unity–diversity models of EFs highlight both shared and separable executive components, such as inhibition, WM updating, and shifting across individuals and tasks, which change with age and cognitive maturation (Duncan & Friedman, 2024; Miyake et al., 2000; Silva et al., 2024). Contemporary work on MW similarly emphasises that MW is not a unitary construct; instead, it comprises intentional (deliberate) and unintentional (spontaneous) forms, which exhibit different cognitive correlates and can be differentially affected by task demands and control processes (Seli et al., 2016). Developmentally, younger children’s EFs are less differentiated and may be more prone to unintentional, control-failure episodes of MW, whereas adolescents and young adults show a greater capacity for intentional, goal-directed MW (reflecting emerging meta-cognitive and motivational dynamics) alongside continued involuntary lapses. This framework suggests that age-related changes in both EF structure and the content/intentionality of MW help explain why some measures (e.g., physiological state indices) capture EF–MW coupling more consistently than broad trait reports, and why associations vary systematically with age and methodology.

The neural correlates of EFs are largely located in the frontal regions of the brain (Korzeniowski et al., 2021). Synaptic pruning occurs in the prefrontal cortex, which has been linked to EFs (Kolk & Rakic, 2022), and is associated with reduced synaptic density and variation in grey matter in the brain during childhood and adolescence (Korzeniowski et al., 2021). The grey matter volume peaks in adolescence and then declines (Korzeniowski et al., 2021). The different dimensions of EFs tend to display a developmental spurt between 6 and 12 years of age (Hughes, 2011). The above findings seem to suggest that more refined cognitive control is achieved after the end of late adolescence, which might influence the links between EFs and MW. Hence, the current review aims to provide a more developmentally sensitive overview of studies that have explored the links between the core EFs and MW in children and adolescents.

### 1.3. The Present Review

The current systematic review aims to clarify the relationship between the core EFs (i.e., working memory, inhibitory control, and cognitive flexibility) and MW (e.g., daydreaming, task-unrelated thoughts, off-task thinking, etc.) in children and adolescents. The purpose of the systematic review is to highlight our current knowledge in this area in younger populations, the various methodologies that have been adopted, and the potential directions for future research in this area of scientific inquiry. This is an important topic because MW may reflect a potential lapse in attention control and EFs in general (Keulers & Jonkman, 2019). Evaluating the evidence regarding the potential link between EFs and MW can provide new insights into the factors contributing to MW and help guide future research in determining whether and how EFs in children and adolescent populations can lead to instances of MW in key domains of children’s and adolescents’ life, such school and university environments. The following research questions guided the review:

- RQ1: What is the association between EFs and MW?
- RQ2: Which EFs were most strongly associated with MW?
- RQ3: What kind of measures were utilised in the measurement of EFs and MW?
- RQ4: What are the limitations of previous approaches and what future directions for research have arisen from previous studies?

## 2. Materials and Methods

### Keyword Search Criteria in Databases

Several keywords were utilised to search databases to identify records; they included MW and the three core EFs. Synonymous terms for MW and the core EFs were employed in the Boolean search strings to ensure that the searches return as many relevant hits as possible. The inclusion criteria were as follows:

- A combination of at least one core EF and one aspect of MW.
- Child or adolescent populations (up to 20 years old (Sawyer et al., 2018)).
- Quantitative studies.
- Peer-reviewed journal papers.
- English-language papers.
- The studies can use behavioural or self-report methodologies but not brain imaging.

The review only focused on published peer-reviewed papers to ensure that the results were more robust compared to the grey literature that has not undergone systematic peer review. Grey literature was excluded in this review to ensure that only peer-reviewed studies with established methodological standards were included, thereby enhancing the reliability and validity of the reviewed studies.

Detailed information about the keyword strings used and the filters applied, as well as the number of retrieved sources from the database searches is presented in Table 1. The literature search was not restricted by publication year in order to maximise inclusivity and capture the full breadth of the relevant literature. Searches were conducted across all databases from their inception up to 1 October 2025, which represents the final date on which the searches were executed. This systematic review was not prospectively registered.

## 3. Results

### 3.1. Study Selection

Following the searches of the databases, a large number of studies that met the inclusion criteria outlined above were identified. Several duplicate references were identified and removed (duplicate removal process) based on title and doi searches, leaving 444 viable articles. These articles were further screened based on their title and abstract to ensure that they met the specified inclusion criteria.

**Figure 1:**
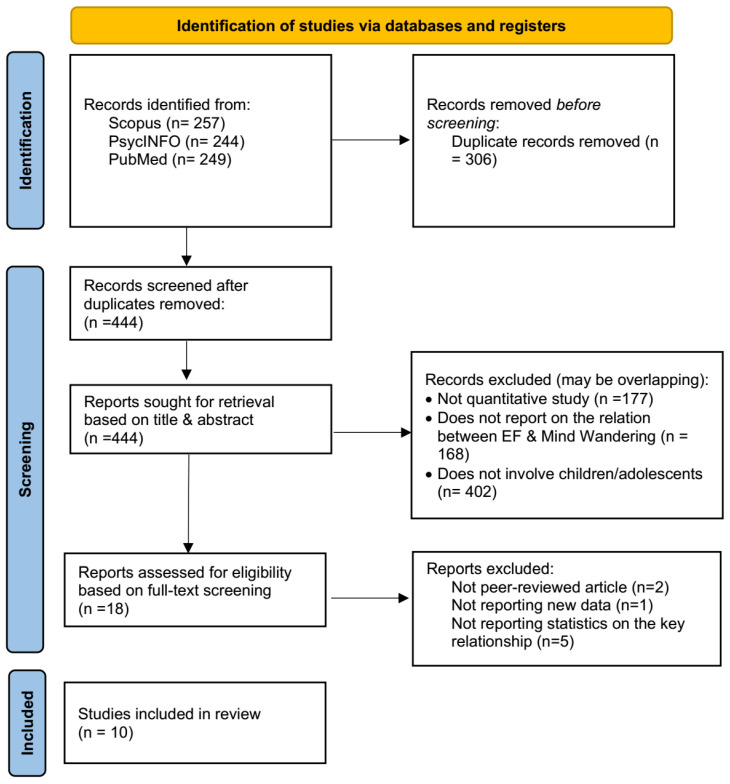
PRISMA flowchart explaining the selection and exclusion of studies.

Following the duplicate removal process, all the remaining articles were screened in two stages: first, the records were screened based on their title and abstract and second, the articles were evaluated for full-text access eligibility, following the PRISMA guidelines. At the title/abstract stage, studies were deemed eligible for exclusion if they failed any of the pre-specified inclusion criteria (i.e., explicitly reported quantitative evidence on the relationship between EFs and MW; the sample comprised children/adolescents (average age up to 20 years old); the study was a peer-reviewed journal study; the article was written in English; and the study did not only use neuroimaging). Since the studies could fail multiple eligibility criteria, the exclusion counts for the different criteria in the title/abstract screening may overlap. Overall, 18 studies were retained, of which, only 10 were selected for in-depth full-text screening and further discussion. The selection of studies for the current review is summarised in the Preferred Reporting Items for Systematic Reviews and Meta-Analysis (PRISMA) flowchart (Page et al., 2021) in Figure 1. The PRISMA checklist is presented in the Supplementary Materials.

### 3.2. Charting the Data

Following the suggestions of (Cooper et al., 2019), the final 10 studies included in the review were read in full to extract key information, such as the sample composition, the aims of the study, the methodology and the measures utilised, along with the key findings that are relevant for the current systematic review. All data were coded by the first authors and the second author and cross-checked for accuracy. Any disagreements between the two authors were discussed and resolved before the final coding of the data was completed. Details about the studies examined in this review are presented in Table 2.

Twenty percent of the studies utilised questionnaire methods to collect and analyse the data (McBurnett et al., 2014; Zhou et al., 2024). Forty percent of the studies employed pupillometric and/or eye-tracking methods to collect data about the attention span of the participants in addition to EF and MW data (Hood et al., 2022; Robison & Unsworth, 2019; Unsworth et al., 2019; Unsworth & Robison, 2017). Furthermore, another 40% of the included studies used a mix of computerised behavioural tasks (i.e., reaction times) along with self-report questionnaires and thought probes to examine potential MW during task administration (Hasan et al., 2024, 2025; Keulers & Jonkman, 2019; Wilson et al., 2022).

Fifty percent of the studies were conducted on adolescent samples and the other fifty percent of the studies focused on child samples. Interestingly, the four out of the five studies with adolescent samples employed pupillometric/eye-tracking measures to investigate MW via task-evoked pupillary responses and the potential impact of EFs (Hood et al., 2022; Robison & Unsworth, 2019; Unsworth et al., 2019; Unsworth & Robison, 2017). All the studies using adolescent samples comprised typically developing adolescents who usually studied at colleges or universities (Hood et al., 2022; Robison & Unsworth, 2019; Unsworth et al., 2019; Unsworth & Robison, 2017; Zhou et al., 2024). In the studies with younger samples, one study included children who had diagnoses of ADHD (McBurnett et al., 2014), whereas the other four studies included children who were typically developing without any specific neurodevelopmental disorder (Hasan et al., 2024, 2025; Keulers & Jonkman, 2019; Wilson et al., 2022).

### 3.3. Quality Appraisal

To evaluate the quality of the ten included studies, we applied the Joanna Briggs Institute’s Critical Appraisal Checklist for cross-sectional studies (Joanna Briggs Institute, 2020) since these ten studies used a cross-sectional methodology. Overall, the reviewed studies met most JBI criteria, with the most common limitations being related to confounding factor identification/handling, as shown in Table 3. Beyond the JBI appraisal checklist, we explain in detail the methodological biases and measurement limitations by type of methodology (i.e., self-report, pupillometry, and behavioural).

Regarding the self-report questionnaire-based studies, several methodological issues should be noted. The use of questionnaires (e.g., McBurnett et al., 2014; Zhou et al., 2024) that collect information from the same source (i.e., parent, teacher, or self-reports) are prone to common method bias/variance (Podsakoff et al., 2024). The use of self-report probes of MW in child samples may not be the most appropriate method because there is evidence showing that young children (9 years old) experience developmental constraints in introspecting and articulating on their subjective memory states (Ghetti et al., 2011). Behavioural measures, such as those used in some reviewed studies (Hasan et al., 2025; Wilson et al., 2022), tend to provide more accurate and bias-free indices of EFs compared to questionnaire measures (McCoy, 2019). Finally, the pupillometry methods employed in many studies on EFs and MW, particularly with adolescents (Hood et al., 2022; Robinson et al., 2019; Unsworth & Robison, 2017), are characterised by methodological limitations such as the issue of whether baseline pupil diameters reflect a single construct (Ayasse & Wingfield, 2020) and the effects of the background and room luminance as potential confounding factors (Kelbsch et al., 2019). All the above are considered potentially constraining factors when interpreting the findings of a study.

### 3.4. Synthesis of Findings

In this section, we are going to explore the findings from the ten studies that met the inclusion criteria and were deemed of good quality. In our review of correlational evidence, we used the commonly accepted cut-offs of correlations of *r* or rho = 0.10, 0.30, and 0.50 to represent small, medium, and large effect sizes (Cohen, 1988). In the following narrative synthesis, we begin with the studies using child samples (ages 7–10 years old) and then proceed to review the evidence for adolescents (mean age up to 20 years old).

#### 3.4.1. Findings from Studies with Children

One study, who provided evidence from typically developing children in the Netherlands, administered computerised EF tests along with self-report thought probes to examine the link between EFs and MW (Keulers & Jonkman, 2019). The study examined the link using correlations between the three core EFs (WM, cognitive flexibility, and inhibition) and MW based on measurements of task-unrelated thinking before, during, and after the computerised task (Keulers & Jonkman, 2019). The findings indicated that higher task switching costs RT was weakly (*r* = 0.28) associated with total task-unrelated thoughts (Keulers & Jonkman, 2019). WM, measured via the running digit span, was not associated with MW (Keulers & Jonkman, 2019). In contrast, worse performance in the Flanker task (Eriksen & Eriksen, 1974), measured as the interference score, was moderately associated (*r* = 0.33) with task-unrelated thoughts in the classroom listening task and total occurrence of task-unrelated thoughts (*r* = 0.28) (Keulers & Jonkman, 2019). Another study from Canada with typically developing children examined children’s MW via their response patterns in the Metronome Response Task (Wilson et al., 2022). The study found that the children remained focused on the task more often than they experienced MW (Wilson et al., 2022). An increase of 1 standard deviation in the index of behavioural dysregulation, which measures attention shifting, inhibitory control, and emotional control, was linked with 1.09 more occurrences (in standard deviations) of MW (Wilson et al., 2022). At the same time, there was no evidence that the metacognitive difficulties index, which measures WM, planning, organisation, and monitoring, was associated with MW (Wilson et al., 2022).

Two additional studies reported on the relationship between EFs and MW using a combination of computerised experimental tasks and self-report thinking probes. The study by (Hasan et al., 2024) focused on typically developing children in Canada. The authors of the study showed that WM capacity was predictive of fewer MW instances but only for twelve-year-old children and not for younger children in the sample (Hasan et al., 2024). Inhibitory control and task switching were not statistically related to MW (Hasan et al., 2024).

In a follow-up study (Hasan et al., 2025), the same group of authors compared the performance of a subsample of the typically developing children in the previous study (Hasan et al., 2024) with children with an ADHD diagnosis (Hasan et al., 2025). In the pooled sample, WM capacity, task switching, and inhibition were not statistically significantly associated with MW instances (Hasan et al., 2025). In a follow-up regression analysis, the authors showed that WM capacity was associated with less MW in the group of children with high hyperactivity/impulsivity, but not the group of children with low hyperactivity/impulsivity (Hasan et al., 2025). Inhibitory control and task switching did not correlate with the severity of ADHD symptoms in the prediction of MW (Hasan et al., 2025).

Finally, the study by (McBurnett et al., 2014), using a sample of 165 children with an ADHD diagnosis in the US, sought to examine the possibility of revising and re-validating the Kiddie Sluggish Cognitive Tempo (SCT) questionnaire (Becker & Barkley, 2018). Parents and teachers reported on their children and students’ SCT, respectively (McBurnett et al., 2014). This study found that WM difficulties, as reported by the teachers and parents, were moderately positively correlated with daydreaming (McBurnett et al., 2014).

#### 3.4.2. Findings from Studies with Adolescents

Amongst the final selected studies with adolescents, two were based on survey-style questionnaires. The study by Zhou et al. (2024) used a large sample of 601 adolescents from China to estimate a serial mediation model whereby childhood adversity predicted cognitive flexibility, which, in turn, predicted habitual tendencies and MW. MW was measured via two subscales, namely deliberate MW and spontaneous MW (Zhou et al., 2024). At the same time, cognitive flexibility was measured via control and alternative explanations of life occurrences (Zhou et al., 2024). The results of the hierarchical regression showed that the control component of cognitive flexibility consistently predicted both spontaneous (β = −0.28) and deliberate (β = −0.40) MW (Zhou et al., 2024).

Further evidence on the association between MW and EFs comes from studies that tracked the pupillary reactions of adolescent participants. The study by (Unsworth & Robison, 2017) used an adolescent sample to estimate the relationship between baseline and task-evoked pupillary reactions and WM capacity, attention control, and MW. Using structural equations, the authors of the study reported that a higher WM capacity was weakly (*r* = −0.25) associated with fewer instances of off-task thinking, whereas attention control, a subcomponent of inhibitory control (Miyake & Friedman, 2012), was not associated with off-task thinking (*r* = −0.10) (Unsworth & Robison, 2017). In a follow-up analysis, the study reported that when WM capacity and attention control were allowed to load on a superordinate executive attention factor, this new factor was strongly predicted by off-task thinking (β = −0.57) (Unsworth & Robison, 2017). The study highlights that off-task thinking predicts specific aspects of EFs. In another study using an adolescent sample by the same authors (Robison & Unsworth, 2019), the authors aimed to investigate variability in WM capacity and its correlation with pupillary reactions and self-report probes for MW. In this study, it was demonstrated that pupil diameter was a robust indicator of WM capacity and self-reported probes of attentional lapses (e.g., MW, external distraction, absentmindedness) were followed by worse WM capacity (Robison & Unsworth, 2019).

Another study by the same group of authors collected a sample of adolescents and used similar pupillometric techniques to examine associations between oculometric indices and cognitive abilities, personality, and self-reported MW (Unsworth et al., 2019). Correlational analyses revealed that WM capacity did not correlate with self-reported off-task thinking (*rho* = −0.08) nor the indices of MW questionnaires (i.e., deliberate or spontaneous MW) (Unsworth et al., 2019). In contrast, attention control was modestly but statistically significantly related to off-task thinking (*rho* = −0.38) but not with the MW questionnaire responses (*rhos* ≤ 0.10) (Unsworth et al., 2019). For the most part, the eye measures either did not exhibit statistically significant correlations or demonstrated very weak—practically insignificant—correlations with cognitive ability measures (WM capacity and attention control) and the MW questionnaire responses (Unsworth et al., 2019). The correlation between baseline pupil diameter and self-reported off-task thinking reached statistical significance but was rather small (*rho* = 0.17) (Unsworth et al., 2019). It should be noted that the correlations reported were of a non-parametric nature. Finally, another pupillometric research study, using adolescent university students, aimed to investigate the degree of attention control in antisaccade and prosaccade trials and the association with task-unrelated thoughts and WM capacity (Hood et al., 2022). The study reported that a larger pupil diameter was associated with more MW instances in antisaccade trials (Hood et al., 2022). Additionally, greater pupil dilation was associated with a lower WM capacity in the first three seconds during the task; however, WM capacity did not demonstrate statistically significant correlations with preceding antisaccade or prosaccade task-unrelated thinking (Hood et al., 2022). It should be noted that the study ran multiple *t*-tests (Hood et al., 2022), which increases the risk of inflated type I errors (Tabachnick & Fidell, 2012).

#### 3.4.3. Grouping by Study Type and Robustness of Effect Sizes

Across the ten included studies, the strength and consistency of relationship between EFs and MW varied based on study type and measurement approach. In studies relying primarily on self-report or informant-report questionnaires, the reported effect sizes of the associations between EFs and MW were small to moderate (e.g., rs or βs in the range of approximately 0.20–0.40). For example, questionnaire-based evidence suggested moderate associations between working memory difficulties and daydreaming in children with ADHD (McBurnett et al., 2014), and between perceived cognitive flexibility and MW tendencies in adolescents (Zhou et al., 2024). However, these effects were derived from single-source reports and often were assumed to reflect trait-like tendencies, limiting conclusions about momentary attentional lapses. Moreover, the effect sizes in questionnaire studies were not always replicated across reporters or subscales, suggesting modest robustness.

Studies employing behavioural EF tasks in conjunction with self-report MW probes during or after the task tended to report small-to-moderate correlations, frequently in the range of *r* ≈ 0.20–0.35. In child samples, such effects were often age- or subgroup-dependent, with working memory predicting MW only in older children (Hasan et al., 2024) or depending on symptom severity (Hasan et al., 2025). Inhibitory control showed similarly mixed effects, with some moderate associations (Keulers & Jonkman, 2019; Wilson et al., 2022) but several null findings depending on the age of the children (Hasan et al., 2024, 2025). These patterns suggest that behavioural EF–MW associations in children are fragile and context-sensitive, rather than robust across tasks and ages.

Finally, studies in adolescent samples involving pupillometry showed the widest range of effect sizes, from weak bivariate correlations (e.g., |*r*| < 0.20) to moderate-to-large effects in latent-variable or within-person models (e.g., *β* ≈ −0.50) (Unsworth & Robison, 2017). Notably, associations between physiological indices and MW were more consistently observed for probe-based or state-level MW measures than for questionnaire-based MW scales (Unsworth et al., 2019). However, several of the eye-metric associations were small in magnitude or statistically unstable, and some studies relied on multiple uncorrected comparisons (Hood et al., 2022), indicating that effect robustness varies substantially depending on analytic strategy.

Taken together, the above findings suggest that no study type produced consistently large effect sizes for the relationship between EFs and MW in children and adolescents. This summary of effect sizes underscores the substantial methodological heterogeneity in the EF–MW literature and should serve as a caution against interpreting any single effect as definitive evidence.

## 4. Discussion

The current systematic review sought to collate and evaluate the evidence on the relationship between the core EFs (i.e., working memory, inhibition, and cognitive flexibility) and MW (e.g., daydreaming, task-unrelated or off-task thinking, etc.) in child and adolescent populations. The subobjectives of the review were to summarise the conceptualisation and measurement of the constructs in child and adolescent samples, to evaluate the strength and the direction of the association between EFs and MW, and to pinpoint potential developmental trends and prospects for further empirical research. To address the mixed and heterogeneous findings in the reviewed literature, the present discussion is organised following three complementary theoretical lenses, namely the e executive-failure perspective, the resource-control perspective, and the developmental measurement perspective. These lenses are used to interpret the observed variability across age groups, methods, and core EFs.

### 4.1. Executive-Failure Interpretations of MW–EF Associations

From an executive-failure perspective, mind wandering reflects lapses in goal maintenance and executive control (McVay & Kane, 2010a). Across the methodologies considered here, several consistent patterns were observed. Firstly, the reviewed studies seemed to indicate that a higher WM capacity was generally related to lower rates of task-unrelated or off-task thinking (Hasan et al., 2024, 2025; Hood et al., 2022; McBurnett et al., 2014; Robison & Unsworth, 2019; Unsworth & Robison, 2017). Yet, there was a study on younger children that reported a nonsignificant correlation between WM capacity and MW (Keulers & Jonkman, 2019). Secondly, evidence on the link between MW and inhibitory control could be characterised as rather mixed at best. While several studies demonstrated that better inhibitory control was associated with fewer instances of MW (Hasan et al., 2024; Keulers & Jonkman, 2019; Unsworth & Robison, 2017; Wilson et al., 2022), other studies reported a statistically insignificant correlation (Hasan et al., 2025).

Thirdly, we noted a limited coverage of the links between the cognitive flexibility EF and MW. The few studies that explicitly covered the association between cognitive flexibility and MW showed mixed results. For example, Hasan et al. (2024, 2025) reported statistically insignificant associations between cognitive flexibility and MW, whereas other studies reported a negative association, such that higher flexibility was associated with lower rates of MW (Keulers & Jonkman, 2019; Zhou et al., 2024). Among the pupillometric studies reviewed here, the study by Hood et al. (2022) provides more robust evidence in favour of the relationship between cognitive flexibility and MW. Specifically, the study showed that flexible adjustment of attentional control between antisaccade and prosaccade trials can be captured by adolescents’ cue-evoked pupil responses (Hood et al., 2022). The adolescent participants in that study were capable of adjusting their pupillary arousal or control depending on the task difficulty, which demonstrates cognitive flexibility (Diamond, 2013). Importantly, when the participating adolescents reported MW via the self-report probes, these cue-related adjustments disappeared, which is indicative of disruption in the adaptive adjustment of attention during off-task thinking states.

### 4.2. Resource-Control/Dynamic Regulation Lens

From a resource-control perspective, mind wandering reflects dynamic reallocation of attentional resources rather than simple control failure (Thomson et al., 2015). At this point, we hypothesise that the variation in studies’ findings might be partially attributed to the neurocognitive development of EFs from late childhood to late adolescence. Earlier, it was mentioned that EFs undergo a protracted maturation between the ages of 7 and 20 years old, with notable improvements in WM capacity, inhibitory control efficiency, and the flexible coordination of attentional resources (Hughes, 2011; Korzeniowski et al., 2021). The development of the prefrontal cortex (PFC), which is implicated in executive functioning (Salehinejad et al., 2021), is known to stabilise in adolescence at approximately 12 years old (Kolk & Rakic, 2022). Empirical studies also suggest that late adolescents perform better in EFs tasks compared to children (e.g., (Tervo-Clemmens et al., 2023)). In younger children (approximately 7–10 years), executive control systems are still relatively fragile and less differentiated (Hartung et al., 2020), which aligns with the unity–diversity models of EFs (Silva et al., 2024). At this stage, MW may reflect broad attentional instability or regulatory immaturity rather than failures in a specific EF. This may help explain why associations between WM and MW are rather weak, inconsistent, or age-dependent in child samples, with several studies reporting null or conditional effects that only emerge in older children or specific subgroups (e.g., children with severe ADHD symptoms). In contrast, adolescence is characterised by rapid gains in executive efficiency (Ferguson et al., 2021; Hughes, 2011). During adolescence, MW seems to reflect dynamic fluctuations in executive engagement and arousal, rather than stable structural deficits in inhibition or working memory, which explains why state-level physiological measures show stronger and more consistent associations with MW than trait or task-level EF indicators (Robison & Unsworth, 2019; Unsworth et al., 2019; Unsworth & Robison, 2017).

Some studies’ findings (Hood et al., 2022; Robison & Unsworth, 2019) appear to suggest that the root contributing factor to MW might not be the inhibitory control lapse per se, but a more general flexible attentional control issue. Specifically, (Hood et al., 2022) indicate that adolescents are able to engage or relax their attentional control as needed depending on the cognitive demands of the tasks. Another study (Robison & Unsworth, 2019) suggests that pupil dilation is dynamic and significantly larger during good trials and attentional lapses are not necessarily associated with instances of low arousal or inhibition, which implies that adolescents are adaptively reallocating cognitive resources. These findings are clearly linked with the explanations provided by the resource-control theory of sustained attention (Thomson et al., 2015). The theory mentions that the degree of executive control decreases over time during task processing, due to the reallocation of cognitive resources to MW, which results in reduced task performance (Thomson et al., 2015).

### 4.3. Developmental and Measurement Lens

A developmental and measurement lens helps explain the heterogeneity in the reviewed findings. As described in the Results section, the magnitude of the associations between the different EFs and MW was weak to moderate, with the available coefficients ranging between −0.08 to −0.56. As noted in (Unsworth et al., 2019), few, if any, associations were demonstrated between self-report measures of MW and behavioural measures of EFs. In contrast, another study reported sizable correlations between measures of executive attention control, a measure of EFs, and off-task thinking (Unsworth & Robison, 2017). The above suggests that there might be a discrepancy in the reliability or the validity of the measures employed in the different studies. The differences between the correlations reported by the self-report studies (McBurnett et al., 2014; Zhou et al., 2024) and those provided by the studies using behavioural computerised methodologies (Hasan et al., 2024; Unsworth et al., 2019) indicate that the different methodologies might measure different aspects of MW and/or EFs.

The abovementioned discrepancies in the relationship between EFs and MW can be traced to the fundamentally different ways of operationalising EFs and MW. Questionnaires, either self-report or informant report, are only able to capture trait-like tendencies (Steyer et al., 2015). Consequently, questionnaire-based studies tend to produce more consistent but modest associations, whilst behavioural and physiological studies yield weaker and more variable effects that are highly context-dependent. Moreover, it should be noted that the different methods for probing MW can lead to different conclusions (Kane et al., 2021), which is why it is important to develop more robust methodologies for capturing MW instances.

Regarding the limitations of the reviewed studies, several things should be mentioned. First, the self-report probes and the questionnaires may not have accurately captured children’s and adolescents’ MW instances because of social desirability bias. Second, the computerised behavioural studies did not explicitly control for potentially confounding factors, especially considering the nature of the samples’ collection (e.g., via the available pools of participants at the universities) and the samples’ diverse demographics (i.e., different ethnic compositions). Third, a couple of studies included both typically developing participants and atypically developing participants (e.g., children with ADHD). This mix of participants, in cases where pooled analyses across participants were performed, can lead to conflating the characteristics of a mental health condition and MW that occurs naturally during task processing. Fourth, there is no clear specification of the direction of the association between EFs and MW measures. For instance, the study by (Unsworth & Robison, 2017) specified a structural model, whereby MW measures predicted EFs, whereas other studies estimated the inverse relationship (Hasan et al., 2024; Keulers & Jonkman, 2019; Wilson et al., 2022). Last but not least, many studies included one or two EFs but not all three, which limits the extent of holistic conclusions.

### 4.4. Integrating the Findings in a Conceptual Model

**Figure 2:**
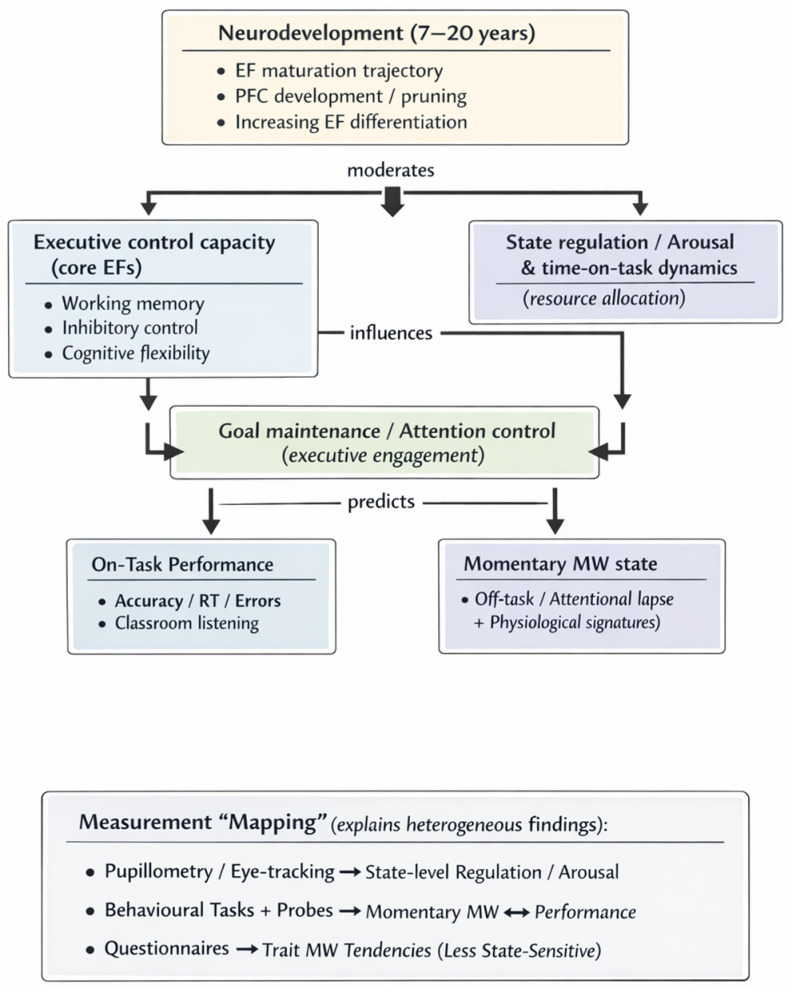
Developmental integrative model for EF–MW relationship in childhood and adolescence. Note: Directionality is not implied and arrows reflect hypothesised pathways consistent with the findings of the reviewed studies.

To integrate the above findings, we constructed a *conceptual* model that might prove useful for future research on MW and EFs. The multimethod integrative model of the EF–MW relationship in childhood and adolescence is graphically presented in Figure 2. Taken together, the executive-failure, resource-control, and developmental and measurement lenses that we outlined above suggest that the EF–MW relationship is neither unitary nor static across development (childhood and adolescence). Instead, MW emerges from interactions between executive capacity, dynamic attentional regulation, and developmental stage. Figure 2 presents an integrative conceptual model illustrating these pathways and clarifies how different methods capture distinct levels of the EF–MW system.

### 4.5. Future Directions for Research and Limitations

In terms of future directions for research, there are several important suggestions that arose from the current systematic review. For instance, we have not been able to find any longitudinal studies that examined the relationship between EFs and MW. Thus, future studies should evaluate the direction of this relationship over time. Moreover, there is a need to provide concrete evidence about the age-related changes in the relationship between MW and EFs during childhood and adolescence. This would answer the key question of, Does the association between MW and EFs differ between adolescents and children? From a methodological point of view, it is also critical to establish whether the computerised behavioural measures and the self-report probes and questionnaires measure similar or diametrically different facets of MW. Further, more research is required in settings other than the US or Canada in order to establish the cross-cultural validity of the evidence in favour of a relationship between EFs and MW. The involvement of neuroscientific insights is also another critical direction going forward so that cognitive scientists can map the neuroscientific links between MW and EFs in children and adolescents. As with all systematic reviews, the current review has a few limitations. For example, we were not able to include studies written in languages other than English, which means that we might have missed some valuable insights. Another limitation is that we only identified a few studies, which made a meta-analysis impossible due to the small sample size and the heterogeneity of the effect. Another key limitation is that the effect varied depending on the method adopted. For instance, self-report studies may overestimate associations due to the common method bias (Podsakoff et al., 2024), whereas behavioural measures might underestimate the associations between EFs and MW due to measurement errors and the use of non-equivalent tasks (Rouder et al., 2023). Future research should prioritise multi-method designs and harmonised EF and MW operationalisation to increase the comparability of studies. Finally, the conceptual model presented above should only be used as a heuristic and is not meant to replace substantive research; rather, future empirical research is needed to verify the validity of the conceptual model.

## 5. Conclusions

In conclusion, this systematic review attempted to explore and synthesise the quantitative evidence on the relationship between EFs and MW in children and adolescents. Taken together, the findings underscore that stronger executive control, particularly WM capacity, seems to be generally associated with fewer instances of MW, such as off-task or task-unrelated thinking, in children and adolescents. The evidence for links between inhibitory control, cognitive flexibility, and MW was rather mixed and scarce in child and adolescent populations. Most importantly, a subset of the behavioural and pupillometric studies suggest that certain facets of MW may not be accurately explained by a lapse in inhibitory control but may reflect flexible, adaptive reallocation of cognitive resources, particularly attention, which aligns well with the resource-control theory (Thomson et al., 2015) and cognitive flexibility frameworks (Beckmann et al., 2024).

At this point, it should be mentioned that the findings of this review hold implications for educational and clinical contexts. However, given the small number of included studies and the substantial heterogeneity in samples and operationalisations, these implications should be regarded as tentative and hypothesis-generating rather than prescriptive. The practical recommendations given below should therefore be interpreted as provisional suggestions based on the reviewed correlational patterns, pending replication and intervention-focused evidence. The reviewed evidence suggests that MW in children and adolescents should not be uniformly interpreted as disengagement or poor self-control. Especially during the adolescent years, MW may often reflect transient fluctuations in executive engagement and arousal rather than stable executive deficits. Consequently, classroom strategies may benefit from avoiding behavioural suppression of off-task behaviour and focusing on adaptive task variation, pacing of the teaching material, and periodic attentional reorientation, which has the potential to better align with adolescents’ developing executive control systems. From an early detection perspective, the findings highlight the limitations of relying exclusively on self-report or informant-report measures of MW and EFs in children. Multi-method screening approaches may, therefore, be more effective for distinguishing between developmental immaturity in EFs, situational EF failure, and clinically relevant executive dysfunction. Nevertheless, the evidence in this area remains limited; thus, multi-method screening should be treated as a promising direction rather than a validated practice guideline. Importantly, the reviewed evidence is predominantly cross-sectional and correlational; therefore, the present findings do not establish that modifying classroom practices or EF-related skills will definitely reduce MW instances.

In conclusion, this review provides several unique contributions. First, this review is, to our knowledge, the first to explore and synthesise the quantitative evidence on the relationship between the core EFs and MW in child and adolescent populations, rather than extrapolating from adult samples. Second, the integration of different methodological approaches to MW and EF measurement highlights that the relationship between EFs and MW can be highly variable depending on the methodology adopted. Third, this review advocates for a developmentally sensitive and informed interpretation of the mixed findings that MW during adolescence often reflects dynamic fluctuations in executive engagement rather than structural executive failure. Finally, the review identified WM as the most consistently executive component associated with MW during early development and highlights the relative paucity of evidence for inhibitory control and cognitive flexibility.

## Figures and Tables

**Table 1:** Keywords, filters, and number of retrieved sources.

Database	Search String (Boolean Logic)	Filters Applied	Number of Hits Retrieved ^1^
PubMed	((“executive function”[MeSH Terms] OR “executive function*”[tiab] OR “working memory”[MeSH Terms] OR “working memory”[tiab] OR “inhibitory control”[tiab] OR “response inhibition”[tiab] OR inhibition[tiab] OR “cognitive flexibility”[tiab] OR “set shifting”[tiab]) AND (“mind wandering”[tiab] OR “spontaneous thought”[tiab] OR “task-unrelated thought”[tiab] OR “off-task thought”[tiab] OR “daydreaming”[tiab])) AND (English[lang]) NOT (“review”[Publication Type] OR “case report”[Publication Type] OR “qualitative”[tiab] OR “meta-analysis”[Publication Type])	English language, journal article only, empirical	250
PsycINFO	(DE “Executive Function” OR “executive function*” OR “working memory” OR “inhibitory control” OR “response inhibition” OR “cognitive flexibility” OR “set shifting”) AND (“mind wandering” OR “task-unrelated thought*” OR “spontaneous thought*” OR “off-task thought*” OR “daydreaming”) AND (quantitative OR “statistical analysis” OR correlation OR regression OR experiment* OR “survey” OR “questionnaire” OR “structural equation model*” OR “reaction time” OR “behavioral measure*” OR “performance task”) NOT (DE “Qualitative Research” OR qualitative OR “case study” OR “review”)	English language, journal article only, empirical	245
Scopus	TITLE-ABS-KEY((“executive function*” OR “working memory” OR “inhibitory control” OR “response inhibition” OR “cognitive flexibility” OR “set shifting) AND (“mind wandering” OR “spontaneous thought*” OR “task-unrelated thought*” OR “off-task thought*” OR “daydreaming”) AND (quantitative OR “statistical analysis” OR correlation OR regression OR experiment* OR “structural equation model*” OR “reaction time” OR “behavioral measure*” OR “performance task”))	English language, journal article only, empirical	258

Note: ^1^ Hits retrieved before the application of filters.

**Table 2:** Details about the ten studies examined in greater detail.

Study	Study Population	Aim(s) of the Study	Methodology	Outcome Measures	Important and Relevant Results
(Robison & Unsworth, 2019)	Adolescents, university undergraduates (mean age: 19 years)	To examine the associations between WM capacity and pre-trial and task-evoked attention span and task-unrelated thoughts.	Behavioural tasks + pupillometry + self-report; correlational and time-series	WM task accuracy, pre-trial pupil diameter, task-evoked pupil dilation, on/off task probes	WM fluctuations co-occurred with attention span lapses. Smaller pupil diameter and smaller task-evoked responses reflected lapses of goal-directed attention.
(Hood et al., 2022)	Adolescents, university undergraduates	To examine whether explicit reminders reduce the WM–Stroop task performance lapses.	Behavioural task + pupillometry + self-report; mixed factorial design	Stroop interference; WM capacity span	Explicit goal reminders reduced the correlation between WM and Stroop-task failure, showing that MW reflects a failure of executive control.
(McBurnett et al., 2014)	Children (mean age: 8.66 years old)	To validate and refine the structure of the Sluggish Cognitive Tempo (SCT) task and examine the distinctness of the SCT factors from ADHD symptoms.	Parent and teacher reports; exploratory + confirmatory factor analyses	Parent and teacher-report on the Sluggish Cognitive Tempo task and on ADHD symptoms	Three-factor structure of the SCT task comprising daydreaming, working memory lapses, and sleepiness/tiredness. SCT and ADHD are related yet distinct constructs.
(Zhou et al., 2024)	Adolescents, college students (mean age: 19.37 years old)	To examine whether early-life adversity predicts MW and the mediating effect of executive functions in the relationship between early-life adversity and MW.	Self-report scales; serial mediation using SEM	Childhood Trauma Questionnaire; Cognitive Flexibility Inventory; Creature of Habit Scale; MW (deliberate and spontaneous)	Control facet of cognitive flexibility negatively predicted mind wandering. Childhood adversity predicted lower perceived control, which predicted more automatic behaviour, which predicted more mind wandering.
(Unsworth et al., 2019)	Adolescents, university students (mean age: 19.09 years old)	To examine how baseline oculometric measures relate to EFs, attention control, personality, and MW.	Eye-tracking + behavioural EF tasks + self-report; correlational modelling	Eye metrics, working memory and attentional control tasks, MW, and personality measures	Higher WM, better attention control, and less MW were associated with larger pupil diameter.
(Keulers & Jonkman, 2019)	Children (mean age: 10.12 years old)	To examine MW under two conditions: computerised EF task and real-world classroom listening task. To explore links between specific facets of EFs and MW.	Behavioural EF tasks + classroom listening task + self-report probes; cross-context correlational design	Questionnaires on attention-related cognitive errors and mindfulness attention scale; behavioural computerised EF task	MW frequency was similar between computerised and real-world classroom tasks. Inhibition strongly predicted MW. WM was not related to MW. Poor attention switching predicted more MW.
(Unsworth & Robison, 2017)	Adolescents, university undergraduates (mean age: 19.48 years old)	To explore how WM and executive attention control relate to off-task thinking and physiological indicators.	Behavioural EF battery + pupillometry + self-report; latent-variable SEM	Executive attention and WM tasks; self-report probes and pupillometric measures	Off-task MW states were related to slower reactions times, reduced accuracy rates, smaller pupil diameters. Off-task thinking predicted lower attention control and WM.
(Hasan et al., 2025)	Children (mean age: 9.98 years)	To explore the association between MW and EFs in children with ADHD	Behavioural EF battery + self-report; cross-sectional; regression analyses	Questionnaires on general daydreaming; self-report probes and computerised task	Children with more severe ADHD symptoms and higher WM capacity had few instances of MW. This result was not robust to multiple comparison correction.
(Hasan et al., 2024)	Children (mean age: 10.06 years)	To explore the relationship between MW and EFs in children (8 to 12 years old)	Behavioural EF battery + self-report; cross-sectional and regression analyses	Questionnaires on MW; self-report probes and computerised task	12-year-olds with a greater WM capacity exhibited a lower frequency of MW. Greater inhibitory control was negatively correlated with MW in the 12-year-olds, whereas task switching did not interact with age in predicting MW.
(Wilson et al., 2022)	Children (mean age: 7.64 years)	To examine the link between MW during the Metronome Response Task and estimate the relationship between executive dysfunction and MW.	Behavioural EF battery + self-report + parent-report; cross-sectional; multilevel modelling	Questionnaire on executive dysregulation; self-report probes and computerised task	More frequent reports of being on-task rather than MW. Inhibition difficulties, but not WM difficulties, predicted more frequent MW.

Note: WM: working memory; MW: mind wandering; EFs: executive functions; SEM: structural equation modelling.

**Table 3:** JBI Critical Appraisal Checklist for the ten included studies.

	(Robison & Unsworth, 2019)	(Hood et al., 2022)	(McBurnett et al., 2014)	(Zhou et al., 2024)	(Unsworth et al., 2019)	(Keulers & Jonkman, 2019)	(Unsworth & Robison, 2017)	(Hasan et al., 2025)	(Hasan et al., 2024)	(Wilson et al., 2022)
Were the criteria for inclusion in the sample clearly defined?	Yes	Yes	Yes	Yes	Yes	Yes	Yes	Yes	Yes	Yes
Were the study subjects and the setting described in detail?	No	Yes	Yes	Yes	Yes	Yes	Yes	Yes	Yes	Yes
Was the exposure measured in a valid and reliable way?	Yes	Yes	Yes	Yes	Yes	Yes	Yes	Yes	Yes	Yes
Were objective, standard criteria used for measuring the condition?	Yes	Yes	Yes	Yes	Yes	Yes	Yes	Yes	Yes	Yes
Were confounding factors identified?	Partly	Partly	Partly	Partly	Partly	Partly	Partly	Partly	Partly	Partly
Were strategies to deal with confounding factors stated?	Partly	Partly	Partly	Partly	Partly	Partly	Partly	Partly	Partly	Partly
Were the outcomes measured in a valid and reliable way?	Yes	Yes	Yes	Yes	Yes	Yes	Yes	Yes	Yes	Yes
Was the appropriate statistical analysis used?	Yes	Yes	Yes	Yes	Yes	Yes	Yes	Yes	Yes	Yes

Note: Quality assessment criteria were obtained from the Joanna Briggs Institute (Joanna Briggs Institute, 2020).

## Data Availability

Not applicable.

## Supplementary Materials

The following supporting information can be downloaded at: https://www.mdpi.com/article/10.3390/jintelligence14020020/s1, PRISMA Checklist.

## Abbreviations

The following abbreviations are used in this manuscript:

ADHD	Attention Deficit Hyperactivity Disorder
WM	working memory
MW	mind wandering
EFs	executive functions
SEM	structural equation modelling
RT	reaction time
